# Excess Frequent Insufficient Sleep in American Indians/Alaska Natives

**DOI:** 10.1155/2013/259645

**Published:** 2013-02-21

**Authors:** Daniel P. Chapman, Janet B. Croft, Yong Liu, Geraldine S. Perry, Letitia R. Presley-Cantrell, Earl S. Ford

**Affiliations:** Division of Population Health, National Center for Chronic Disease Prevention and Health Promotion, Centers for Disease Control and Prevention, 4770 Buford Highway NE, Mailstop K-67, Atlanta, GA 30041, USA

## Abstract

*Objective*. Frequent insufficient sleep, defined as ≥14 days/past 30 days in which an adult did not get enough rest or sleep, is associated with adverse mental and physical health outcomes. Little is known about the prevalence of frequent insufficient sleep among American Indians/Alaska Natives (AI/AN). *Methods*. We assessed racial/ethnic differences in the prevalence of frequent insufficient sleep from the combined 2009-2010 Behavioral Risk Factor Surveillance Survey among 810,168 respondents who self-identified as non-Hispanic white (NHW, *n* = 671,448), non-Hispanic black (NHB, *n* = 67,685), Hispanic (*n* = 59,528), or AI/AN (*n* = 11,507). *Results*. We found significantly higher unadjusted prevalences (95% CI) of frequent insufficient sleep among AI/AN (34.2% [32.1–36.4]) compared to NHW (27.4% [27.1–27.6]). However, the age-adjusted excess prevalence of frequent insufficient sleep in AI/AN compared to NHW was decreased but remained significant with the addition of sex, education, and employment status; this latter relationship was further attenuated by the separate additions of obesity and lifestyle indicators, but was no longer significant with the addition of frequent mental distress to the model (PR  =  1.05; 95% CI : 0.99–1.13). This is the first report of a high prevalence of frequent insufficient sleep among AI/AN. These results further suggest that investigation of sleep health interventions addressing frequent mental distress may benefit AI/AN populations.

## 1. Introduction

Recent research indicates that at least one-third of US adults report regularly getting less than the 7–9 hours of sleep per night recommended by the National Sleep Foundation [1]. This finding poses important implications for health and development. Insufficient sleep, variably defined, has been associated with health-risk behaviors, such as smoking [2, 3], alcohol use [2, 3], and with adverse health outcomes such as obesity [4], and frequent mental distress (FMD) (≥14 days/past 30 days in which respondents report their mental health was not good) [3]. Noting that the average sleep duration among US adults has decreased during the past 50 years along with a concomitant increase in the prevalence of obesity, Wheaton et al. [4] found a strong positive relationship between perceived insufficient sleep and body mass index (BMI) among community-dwelling adults. Given the linkage between insufficient sleep and increased BMI, the association between insufficient sleep and diabetes [5] is not surprising. Consistent with these findings, sleep restriction (defined as 5 hours/night for one week) was found to be associated with a significant decrease in insulin sensitivity [6].

Notably, the prevalence of several factors associated with insufficient sleep has been reported to vary between different race/ethnicities in large epidemiologic studies. Specifically, an analysis of 2006–2008 BMI data from the Behavioral Risk Factor Surveillance Survey (BRFSS) revealed prevalences of obesity of 23.7% among non-Hispanic whites (NHW), 35.7% among non-Hispanic blacks (NHB), and 28.7% among Hispanics [7]. Although AI/AN were not specifically delineated in the latter survey, rates of obesity in AI/AN adults have been reported to be 1.5 times greater than among NHW adults [8]. Similarly, aggregated prevalences (1999–2004) of diagnosed diabetes reported from the National Health and Nutrition Examination Survey (NHANES) were 5.9% among NHW, 11.1% among NHB, and 10.9% among Mexican Americans [9]. An analysis of data from both the BRFSS and the Indian Health Service across 1994–2002 revealed that the age-adjusted prevalence of diabetes among AI/AN adults was more than twice that of US adults overall [10]. 

Despite the relatively high prevalence of factors associated with insufficient sleep in AI/AN, few epidemiologic investigations of insufficient sleep have been conducted in this population. In investigations delineating race/ethnicity disparities, short sleep duration (average ≤6 hours/24-hour period) among workers was reported significantly more frequently by NHB (38.9%), NH other (35.3%), and NH Asian (33.2%) than among NHW (28.6%) or Hispanic (28.8%) [11]. In related research using NHANES 2005–2008 data to assess sleep-related difficulties (e.g., concentrating, remembering, driving, and working), NHB reported a greater prevalence of sleep-related difficulties in driving or taking public transportation (14.8%) than the other racial/ethnic populations studied (NHW, Mexican Americans, and others) [1]. Similarly, reporting 30 days of insufficient sleep or rest was significantly more prevalent among NHB (13.3%) than NHW (11.2%) 2008 BRFSS respondents [12]. It is worthy of note that because of small sample sizes of AI/AN in national surveillance systems, AI/AN are often categorized as “others,” thus rendering it difficult to ascertain the prevalence of sleep sufficiency and risk factors in this population. 

As many risk factors associated with frequent insufficient sleep appear to be of increased prevalence in AI/AN, we sought to determine the prevalence of insufficient sleep in a community-based sample of this population. Specifically, to examine if AI/AN are more likely to experience insufficient sleep relative to NHW, as has already been reported among NHB [12], we aggregate data from the 2009 and 2010 BRFSS. Furthermore, we assess the impact of recognized sleep correlates (i.e., socioeconomic indicators, lifestyle behaviors, obesity, age, and FMD) on potential race/ethnicity disparities in insufficient sleep.

## 2. Methods

Data were obtained from the recent BRFSS, a large, random-digit-dialed telephone survey conducted in all 50 states, the District of Columbia, and US territories. The 2009-2010 BRFSS collected data on health-related behaviors, including sleep, smoking, physical inactivity, binge drinking, obesity, and frequent mental distress among the US civilian population aged ≥18 years living in households with landline telephones. Trained interviewers administered standardized questionnaires to households selected through a disproportionate stratified sample design. As previously noted, we combined 2009 and 2010 BRFSS data in order to yield an adequate sample size of AI/AN. Before aggregating these data, we observed no difference between 2009 and 2010 in frequent insufficient sleep, defined as ≥14 days/past 30 days in which respondents reported that they did not get enough rest or sleep. Additionally, the two years shared a similar median response rate to the question assessing frequent insufficient sleep (52.5% and 54.6%, respectively) (BRFSS 2009 and 2010 Summary Quality Report, version 1, revised on 2/18/2011 for 2009 and on 5/2/2011 for 2011). A detailed description of the BRFSS survey design, data collection techniques, and the full-text questionnaire can be found at http://www.cdc.gov/brfss.

Data were obtained from 810,168 respondents (96.9%) who self-identified as NHW, (*N* = 671,448), NHB (*N* = 67,685), Hispanic (*N* = 59,528), or AI/AN (*N* = 11,507) after excluding respondents who had missing data on the insufficient sleep question (*n* = 15,322) and other variables of interest (*n* = 25,636).

### 2.1. Measures

#### 2.1.1. Frequent Insufficient Sleep

All respondents were asked, “During the past 30 days, for about how many days have you felt you did not get enough rest or sleep?” We defined frequent insufficient sleep as ≥14 days, as this cutoff has been shown to have a strong relationship with the prevalence of chronic disease and health risk behaviors [3].

#### 2.1.2. Covariates

Sociodemographic characteristics included sex, age in years (18–24, 25–34, 35–44, 45–54, 55–64 or ≥65), years of education (<12, 12, or >12), and employment status (employed for wage/self-employed, unemployed, retired, unable to work, or homemaker/student).

Health-related lifestyle behaviors included smoking status (current smoker, former smoker, or never smoked), binge drinking (for men, ≥5 alcoholic beverages on one occasion in the previous 30 days; for women, ≥4 alcoholic beverages on one occasion in the previous 30 days), and physical inactivity (respondent indicated “no” to the question, “During the past month, other than your regular job, did you participate in any physical activities or exercising such as running, calisthenics, golf, gardening, or walking for exercise?”). Frequent mental distress (FMD) was defined as a response of ≥14 days to the question, “Now thinking about your mental health, which includes stress, depression, and problems with emotions, for how many days during the past 30 days was your mental health not good?” [13]. Assessment of obesity was based on the body mass index (BMI, kg/m^2^), calculated from respondents' self-reported height in inches and weight in pounds (obese: BMI ≥30 kg/m^2^ versus not obese: BMI <30.0 kg/m^2^) [14].

### 2.2. Statistical Analysis

First, the racial/ethnic-specific distributions of the selected characteristics and frequent insufficient sleep were obtained in order to examine unadjusted race/ethnicity disparities. Next, the prevalence of frequent insufficient sleep by race/ethnicity and groups defined by obesity, smoking, binge drinking, physical activity, and FMD status was calculated to demonstrate race/ethnicity-specific associations between these covariates and frequent insufficient sleep. We used multivariate logistic regression modeling to assess the race/ethnicity disparity in frequent insufficient sleep in separate models controlling first for age, then in a second model with the addition of sex, education, and employment, to which obesity was added to create a third model. A fourth model added smoking status, physical inactivity, and binge drinking to the third model, with a fifth model adding FMD. All analyses were conducted using SAS-callable SUDAAN to account for the complex sampling design [15]. Statistical significance was denoted as *P* ≤ 0.01.

## 3. Results

Distributions of selected characteristics by race/ethnicity are shown in Table 1. AI/AN had a higher proportion of male respondents than other racial/ethnicity groups. Relative to NHW, we found that NHB, Hispanics, and AI/AN tended to be younger, to report <12 years of education and were more likely to be unemployed or to report being unable to work (*P* ≤ 0.01). In addition, NHB, AI/AN, and Hispanics were more likely to be obese relative to NHW (*P* ≤ 0.01) and, concomitantly, to report they were physically inactive (*P* ≤ 0.001). AI/AN were more likely to report being current (33.2%) or former (25.7%) smokers than any of the other race/ethnicity groups examined. Binge drinking was similar in all race/ethnicity groups except among NHB who were less likely to report binge drinking. Table 1 also indicates that the unadjusted prevalence of FMD was significantly greater (*P* ≤ 0.001) among AI/AN than any other race/ethnicity group. Most notably, we found significantly higher unadjusted prevalences (95% confidence interval [95% CI]) of frequent insufficient sleep among AI/AN (34.2% [32.1–36.4]) and NHB (29.4% [28.6–30.1]) and a significantly lower prevalence among Hispanics (25.4% [24.7–26.1]) compared to NHW (27.4% [27.1–27.6]). 

Table 2 lists the race/ethnicity-specific prevalences of frequent insufficient sleep by selected characteristics. Obese respondents had a statistically higher prevalence of frequent insufficient sleep than nonobese respondents among all race/ethnicities, excepting AI/AN. Regardless of race/ethnicity, current smokers were more likely to report frequent insufficient sleep than former smokers or never smokers (*P* ≤ 0.01). A higher prevalence of frequent insufficient sleep among binge drinkers compared to nonbinge drinkers reached statistical significance only among NHW and NHB (*P* ≤ 0.01). A positive relationship between physical inactivity and frequent insufficient sleep was observed regardless of race/ethnicity (*P* ≤ 0.01). Persons reporting FMD were significantly more likely to report frequent insufficient sleep than those without FMD in all race/ethnicity groups (*P* ≤ 0.001). 

Results from our adjusted multivariate logistic regression models comparing the likelihood of frequent insufficient sleep among NHB, Hispanics, and AI/AN relative to frequent insufficient sleep in NHW are shown in Table 3. After age adjustment (Model 1) the disparity in frequent insufficient sleep between NHW and NHB (PR = 1.02; 95% CI: 0.99–1.04) was no longer statistically significant. However, both NHB (PR = 0.95; 95% CI: 0.92–0.98) and Hispanics (PR = 0.84; 95% CI: 0.81–0.86) remained significantly less likely (*P* ≤ 0.001) to report frequent insufficient sleep than NHW, even after controlling for all the covariates in the model (Model 5). 

Table 3 also reveals a significant age-adjusted excess prevalence of frequent insufficient sleep in AI/AN relative to NHW in Model 1, (PR = 1.20; 95% CI: 1.12–1.27). This was modestly weakened by the addition of sex, education, and employment (PR = 1.12; 95% CI: 1.04–1.19) as indicated by Model 2. This relationship was further attenuated in AI/AN by the separate addition of obesity in Model 3 (PR = 1.10; 95% CI: 1.03–1.18), and then by the addition of smoking, physical inactivity, and binge drinking (PR = 1.08; 95% CI: 1.01–1.16) in Model 4. Finally, the disparity in frequent insufficient sleep was no longer significant after adding FMD in Model 5 (PR = 1.05; 95% CI: 0.99–1.13). 

## 4. Discussion

This is the first investigation to document the excess prevalence of frequent insufficient sleep among AI/AN relative to other race/ethnicity groups. Over 34% of AI/AN reported frequent insufficient sleep ≥14 days in the past month in contrast to 29% of NHB, 27% of NHW, and 25% of Hispanics. The disparity in frequent insufficient sleep prevalence between AI/AN and NHW remained even after we adjusted for recognized sleep correlates that included sociodemographic characteristics, lifestyle behaviors, and obesity. However, we also found that this disparity was attributable, in part, to the relatively high rate of FMD in the AI/AN population. Notably, previous sleep research in this population has largely been limited to the identification of factors associated with the increased prevalence of sudden infant death syndrome [16–18] and has documented the prevalence of sleep disturbance among AI/AN veterans [19]. 

Other research on sleep health in AI/AN suggests possible implications of the results of the present investigation. Specifically, noting that cardiovascular disease (CVD) is the leading cause of death among AI/AN, Sabanayagam et al. [20] assessed both symptoms of insomnia—including short sleep duration—and self-reported CVD among 449 AI/AN aged ≥55 years of age in the Native Elder Care Study. Notably, these investigators found that relative to participants reporting a 7-hour sleep duration, the multivariable odds ratio for CVD of those with a sleep duration ≤5 hours was 2.89 (95% CI: 1.17–7.16), after adjusting for demographic, lifestyle, and clinical factors [20]. These results suggest that the excess insufficient sleep documented in the present investigation could manifest itself in the increased risk for CVD-related morbidity and mortality in the AI/AN population.

In a review of the literature, Penev [21] posits that insufficient sleep precipitates “metabolic cost,” and triggers a cascade of metabolic, neuroendocrine, and behavioral changes designed to increase food consumption and decrease energy expenditure, thereby giving rise to obesity [22]. The excess insufficient sleep observed in this study may thus underlie findings indicating that AI/AN are at increased risk of obesity relative to other race/ethnicity groups [23–25]. Furthermore, as diabetes is associated with obesity [26–29], our findings may describe a heretofore unrecognized risk factor underlying the increased rate of diabetes among AI/AN [30–32].

 Our results are subject to several limitations. First, our measure of frequent insufficient sleep was based on subjective self-report and is not corroborated by actigraphy or polysomnography. Second, the wording of the insufficient sleep question includes both the terms “rest” and “sleep,” thereby being potentially subject to varying interpretations among respondents. Third, our sample is limited to only households with landline telephones and to respondents residing in noninstitutionalized settings; thus, our findings may not be generalizable to the overall US population.

## 5. Conclusion

Our finding of excess insufficient sleep among AI/AN thus appears to confirm the importance of sleep health to chronic disease prevention and health promotion in this population. Specifically, assessment of sleep sufficiency appears particularly germane to healthcare providers serving this population, as well as to public health messaging targeting sleep health (including sleep correlates) in AI/AN communities. Available interventions designed to promote awareness of the importance of sufficient sleep include a “Summary for Patients” describing the influence of both diet and sufficient sleep on efforts to lose weight [33], as well as cognitive behavioral therapy for insomnia [34, 35], with the latter improving both subjective and objective sleep quality, as measured by polysomnography [36]. Finally, as FMD appears associated with frequent insufficient sleep among AI/AN, mental health assessment and intervention emerge as important components in the evaluation of insufficient sleep in this population.

## Figures and Tables

**Table 1:** Distributions of selected characteristics by race/ethnicity among adults aged ≥18 years, 2009-2010 BRFSS.

Characteristic	NHW (*N* = 671,448) %(95% CI)	NHB (*N* = 67,685) %(95% CI)	Hispanic (*N* = 59,528) %(95% CI)	AI/AN (*N* = 11,507) %(95% CI)
Age, years				
18–24	9.0 ( 8.8–9.2)	12.7 (12.0–13.5)	16.0 (15.3–16.8)	12.8 (11.1–14.6)
25–34	15.6 (15.4–15.8)	19.7 (19.0–20.4)	25.0 (24.3–25.7)	19.4 (17.4–21.4)
35–44	18.8 (18.6–19.0)	21.2 (20.5–21.9)	23.8 (23.1–24.4)	19.9 (18.0–21.8)
45–54	20.3 (20.1–20.4)	19.1 (18.6–19.7)	16.7 (16.2–17.2)	19.0 (17.5–20.4)
55–64	16.2 (16.1–16.4)	14.0 (13.6–14.4)	9.7 ( 9.3–10.1)	15.4 (14.1–16.7)
≥65	20.1 (20.0–20.3)	13.2 (12.8–13.6)	8.8 ( 8.5–9.1)	13.5 (12.3–14.7)
Sex				
Men	48.4 (48.2–48.7)	45.1 (44.3–45.9)	49.7 (48.9–50.5)	55.1 (53.0–57.3)
Women	51.6 (51.3–51.8)	54.9 (54.1–55.7)	50.3 (49.5–51.1)	44.9 (42.7–47.0)
Educational attainment (years)				
<12	6.1 (6.0–6.2)	12.2 (11.7–12.7)	30.8 (30.1–31.5)	18.0 (16.3–19.8)
12	27.6 (27.4–27.8)	33.1 (32.4–33.9)	28.6 (27.9–29.3)	33.2 (31.2–35.3)
>12	66.3 (66.1–66.5)	54.7 (53.9–55.5)	40.6 (39.8–41.4)	48.7 (46.5–50.9)
Employment status				
Employed	58.2 (58.0–58.4)	53.1 (52.3–53.8)	55.6 (54.8–56.3)	49.8 (47.6–52.0)
Unemployed	6.9 (6.8–7.1)	14.3 (13.7–14.9)	12.0 (11.4–12.5)	13.5 (11.9–15.2)
Retired	18.7 (18.6–18.9)	13.6 (13.2–14.0)	7.3 (7.0–7.6)	13.1 (11.8–14.4)
Unable to work	4.6 (4.6–4.7)	9.7 (9.3–10.2)	5.5 (5.1–5.8)	12.7 (11.4–13.9)
Homeworker/student	11.5 (11.3–11.6)	9.3 ( 8.8–9.9)	19.7 (19.1–20.3)	10.9 (9.5–12.3)
Comorbidities				
Obesity (BMI ≥ 30.0 kg/m^2^)	25.3 (25.1–25.5)	37.4 (36.7–38.2)	28.9 (28.2–29.6)	34.3 (32.2–36.4)
Smoking status				
Current smoker	17.9 (17.7–18.1)	19.8 (19.1–20.4)	14.5 (13.9–15.1)	33.2 (31.1–35.4)
Former smoker	28.0 (27.9–28.3)	16.7 (16.2–17.3)	18.1 (17.6–18.8)	25.7 (23.8–27.5)
Never smoked	54.1 (53.8–54.3)	63.5 (62.8–64.3)	67.4 (66.6–68.1)	41.1 (39.0–43.3)
Binge drinking	15.9 (15.7–16.1)	10.2 ( 9.7–10.7)	16.0 (15.3–16.7)	15.7 (13.9–17.6)
Sedentary lifestyle	22.2 (22.1–22.4)	30.9 (30.1–31.6)	30.2 (29.5–30.9)	27.6 (25.7–29.4)
Frequent mental distress (≥14 days/30 days)	10.1 (9.9–10.2)	12.9 (12.4–13.5)	12.0 (11.5–12.5)	17.8 (16.0–19.5)
Frequent insufficient sleep (≥14 days/30 days)	27.4 (27.1–27.6)	29.4 (28.6–30.1)	25.4 (24.7–26.1)	34.2 (32.1–36.4)

**Table 2:** Weighted percentage of frequent insufficient sleep by selected characteristics and race/ethnicity among adults aged ≥18 years, 2009-2010 BRFSS.

Characteristic	NHW (*N* = 671,448) %(95% CI)	NHB (*N* = 67,685) %(95% CI)	Hispanic (*N* = 59,528) %(95% CI)	AI/AN (*N* = 11,507) %(95% CI)
Obesity				
Yes	32.0 (31.6–32.5)	32.7 (31.6–33.8)	30.0 (28.6–31.3)	36.9 (33.0–40.7)
No	25.8 (25.5–26.0)	27.4 (26.5–28.3)	23.4 (22.6–24.1)	32.6 (30.0–35.1)
Smoking status				
Current smoker	38.5 (37.9–39.1)	36.6 (34.9–38.3)	33.2 (31.1–35.4)	45.2 (41.2–49.3)
Former smoker	24.8 (24.4–25.2)	27.1 (25.6–28.7)	26.8 (25.2–28.3)	30.7 (26.9–34.5)
Never smoked	25.0 (24.7–25.3)	27.7 (26.8–28.6)	23.4 (22.6–24.2)	27.3 (24.4–30.3)
Binge drinking				
Yes	31.0 (30.3–31.7)	36.5 (33.8–39.2)	27.2 (25.2–29.3)	38.6 (31.9–45.3)
No	26.7 (26.5–26.9)	28.6 (27.9–29.3)	25.2 (24.5–26.0)	33.4 (31.2–35.7)
Physical inactivity				
Yes	34.7 (34.3–35.2)	33.8 (32.6–35.0)	29.8 (28.5–31.0)	42.8 (39.0–46.7)
No	25.2 (25.0–25.5)	27.4 (26.5–28.2)	23.3 (22.5–24.1)	30.6 (28.0–33.1)
Frequent mental distress (≥14 days/30 days)				
No	23.5 (23.3–23.7)	24.4 (23.6–25.1)	20.3 (19.6–21.0)	26.2 (24.0–28.5)
Yes	60.4 (59.6–61.1)	62.3 (60.2–64.5)	61.3 (59.2–63.4)	70.4 (65.7–75.1)

**Table 3:** Adjusted prevalence ratio (PR) and 95% confidence interval for the association of race/ethnicity to the likelihood of frequent insufficient sleep among adults aged ≥18 years, 2009-2010 BRFSS.

	Model 1	Model 2	Model 3	Model 4	Model 5
	Adjusted PR (95% CI)	Adjusted PR (95% CI)	Adjusted PR (95% CI)	Adjusted PR (95% CI)	Adjusted PR (95% CI)
Race/ethnicity					
NHW	1.00 (referent)	1.00 (referent)	1.00 (referent)	1.00 (referent)	1.00 (referent)
NHB	1.02 (0.99–1.04)	0.95 (0.93–0.98)	0.93 (0.91–0.96)	0.95 (0.92–0.97)	0.95 (0.92–0.98)
Hispanic	0.84 (0.81–0.86)	0.81 (0.79–0.84)	0.81 (0.78–0.83)	0.83 (0.80–0.86)	0.84 (0.81–0.86)
AI/AN	1.20 (1.12–1.27)	1.12 (1.04–1.19)	1.10 (1.03–1.18)	1.08 (1.01–1.16)	1.05 (0.99–1.13)

Model 1: adjusted PR obtained with controlling for age.

Model 2: adjusted PR obtained with controlling for age, sex, education, and employment.

Model 3: adjusted PR obtained with controlling for covariates in Model 2 and obesity (yes versus no).

Model 4: adjusted PR obtained with controlling for covariates in Model 3 and smoking status (current or former smoker versus never smoked), physical inactivity (yes versus no), and binge drinker (yes versus no).

Model 5: adjusted PR obtained with controlling for covariates in Model 4 and frequent mental distress (≥14 days versus <14 days).
